# Alternatives to Antibiotics to Prevent Necrotic Enteritis in Broiler Chickens: A Microbiologist's Perspective

**DOI:** 10.3389/fmicb.2015.01336

**Published:** 2015-12-01

**Authors:** Delphine L. Caly, Romain D'Inca, Eric Auclair, Djamel Drider

**Affiliations:** ^1^Université Lille, INRA, ISA, Université Artois, Université Littoral Côte d'Opale, Institut Charles ViolletteLille, France; ^2^Société Industrielle Lesaffre, Phileo Lesaffre Animal CareMarcq-en-Baroeul, France

**Keywords:** *Clostridium perfringens*, necrotic enteritis, broiler chicken, antimicrobials, probiotic, competitive exclusion, bacteriocin

## Abstract

Since the 2006 European ban on the use of antibiotics as growth promoters in animal feed, numerous studies have been published describing alternative strategies to prevent diseases in animals. A particular focus has been on prevention of necrotic enteritis in poultry caused by *Clostridium perfringens* by the use of microbes or microbe-derived products. Microbes produce a plethora of molecules with antimicrobial properties and they can also have beneficial effects through interactions with their host. Here we review recent developments in novel preventive treatments against *C. perfringens*-induced necrotic enteritis in broiler chickens that employ yeasts, bacteria and bacteriophages or secondary metabolites and other microbial products in disease control.

## Introduction

### *Clostridium Perfringens*, the causative agent for necrotic enteritis

*Clostridium perfringens* is a spore-forming, anaerobic, Gram-positive bacterium, found in the environment and also in the gastro-intestinal (GI) tract of humans and animals (Songer, 1996; Van Immerseel et al., 2004; Popoff, 2013). It is one of the most common causes of foodborne illnesses in humans, but it also poses an important threat for animals (Uzal et al., 2010; Grass et al., 2013). Indeed, *C. perfringens* is responsible for severe infections in animals, such as enterotoxaemia, gangrenous dermatitis and necrotic enteritis (NE), especially in pigs and poultry (Songer, 1996; Van Immerseel et al., 2004; Timbermont et al., 2011).

*C. perfringens* strains can produce up to 17 different toxins (the majors toxins α, β, β2, ε, ι and the enterotoxin CPE), recently reviewed by Uzal et al. (2014). *C. perfringens* isolates are classified in 5 toxinogroups, based on their toxin production (Songer, 1996; Van Immerseel et al., 2009), each set of toxins being responsible for a specific disease (Uzal et al., 2010). For examples, type B strains, which produce α, β, and ε toxins cause lamb dysentery and type D strains, which only produce the α and ε toxins are responsible enterotoxaemia in those animals (Songer, 1996; Uzal et al., 2010, 2014; Popoff, 2013). In poultry, necrotic enteritis is caused mainly by type A strains, which produce the α toxin and the pore-forming toxin NetB (for NE B-like) (Engström et al., 2003; Keyburn et al., 2008; Cooper and Songer, 2009). The α toxin was long thought to be responsible for necrotic enteritis but several reports have since established that the NetB toxin alone can cause the disease (Keyburn et al., 2008, 2010; Van Immerseel et al., 2009).

Another notable mechanism contributing to the virulence of *C. perfringens* is the production of bacteriocins. Virulent strains of *C. perfringens* have been shown to inhibit the growth of other *C. perfringens* strains in order to take advantage during competition for nutrients (Barbara et al., 2008; Timbermont et al., 2009). Recently, Timbermont and colleagues identified perfrin, a novel 11.5 kDa bacteriocin that is produced by a NetB-positive strain isolated from a chicken with NE. Intriguingly, perfrin has no sequence homology to other bacteriocin proteins, suggesting that this is the paradigm for a new class of bacteriocin (Timbermont et al., 2014). It is likely that further bacteriocins remain to be discovered.

### Necrotic enteritis and broiler chickens

*C. perfringens*-induced NE in chickens leads to sudden death, with mortality rates up to 50% (Kaldhusdal and Løvland, 2000; McDevitt et al., 2006; Lee et al., 2011b). More importantly, *C. perfringens* is also responsible for subclinical infections, associated with chronic damage of the intestinal mucosa. Such subclinical infections cause problems such as lower performance and reduced weight gain, which have dramatic economic consequences (Elwinger et al., 1992; Kaldhusdal et al., 2001; Skinner et al., 2010). The cost of NE worldwide was estimated to 2 billion dollars per year, which includes not only direct loss due to broilers deaths, but also veterinary and cleaning costs (Van der Sluis, 2000; Timbermont et al., 2011).

*C. perfringens* is almost always found in healthy chickens, although at levels less than 10^5^ cfu/g intestinal content. The ability of the bacterium to cause disease is linked to several predisposing factors that affect intestinal conditions and create a favorable environment for proliferation. Perhaps the most important of these factors is the incidence of coccidiosis (Al-Sheikhly and Al-Saieg, 1980; Craven et al., 2001; Williams, 2005; Si et al., 2007). NE incidence and the mortality rates are higher when chickens are co-infected with *Eimeria*, a causal agent of coccidiosis (Shane et al., 1985; Baba et al., 1992). The feeding diet has been shown to be another factor favoring disease, through an influence on the properties of the intestinal content such as viscosity and the presence of non-digestible polysaccharides, the GI tract transit time and the intestinal pH (Annett et al., 2002; Drew et al., 2004; Moran, 2014). For example, diets rich in wheat or fish proteins are known to increase the risk of necrotic enteritis (Annett et al., 2002; Drew et al., 2004).

Animals are often infected through bacterial cells or spores present in their feed, from contaminated litter or by cross-contamination with infected animals at the early stages of life. Young animals, which have immature immune systems and no established commensal flora, are primarily at risk. Infected animals show severe lesions of the jejunum and ileum, the small intestine presenting a degenerated mucosa and being distended by gases produced by *C. perfringens*. Signs of infection include the animal looking depressed, moving less and having diarrhea, which is the most visibly obvious symptom. For a more detailed coverage of *C. perfringens* pathogenicity and clinical signs of NE, the reader is directed to a number of other articles (Helmboldt and Bryant, 1971; Van Immerseel et al., 2004; Timbermont et al., 2011; Paiva and McElroy, 2014).

**Figure 1:**
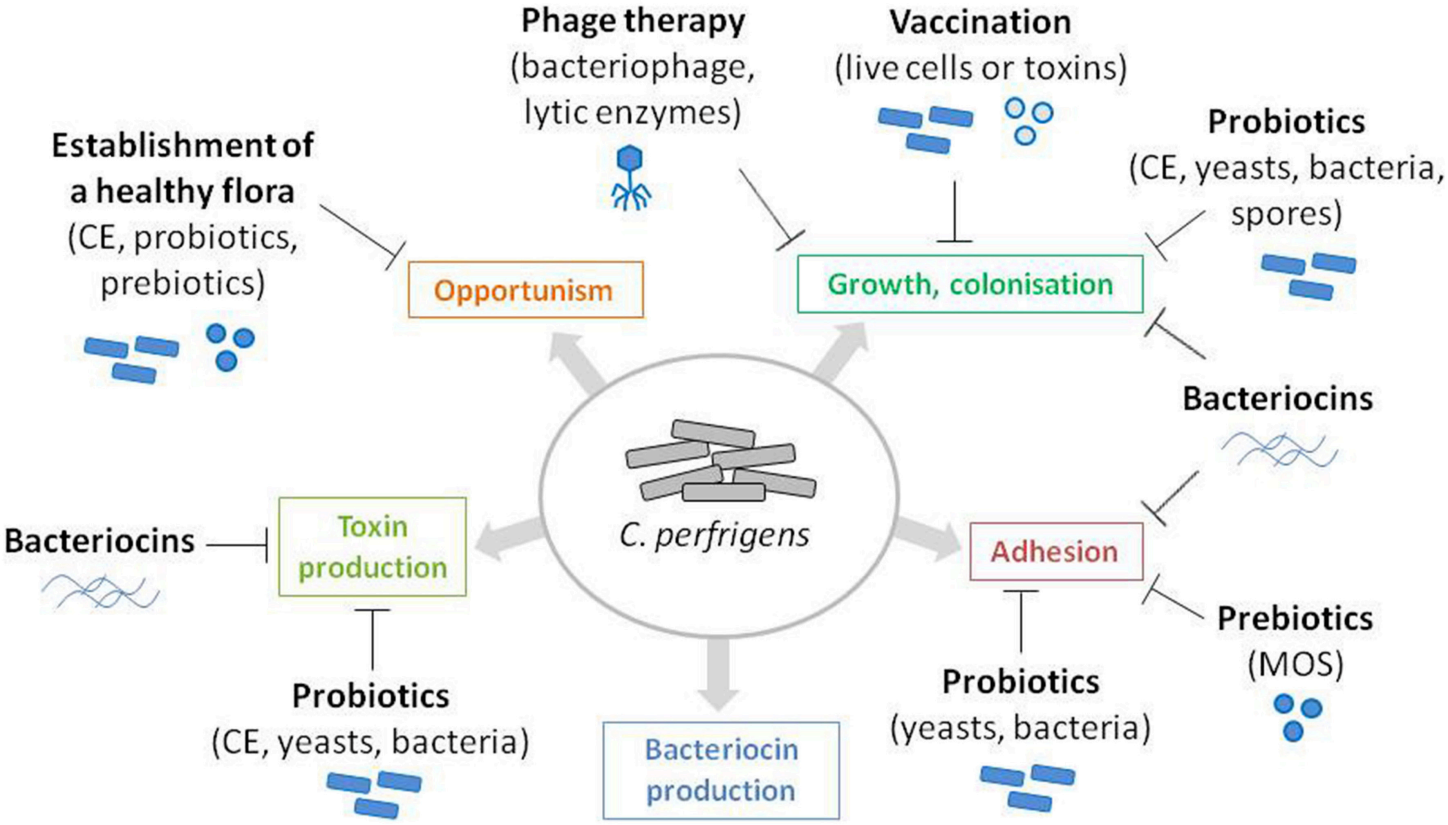
**Identification of ***C***. ***perfringens*** virulence and pathogenicity factors as potential targets for NE prevention**. *C. perfringens* virulence and pathogenicity factors are represented as colored boxes. Antagonistic action of the microbes and microbe-derived products discussed in this review are represented by flat-end arrows.

The rapid death (within 24 h) of chickens with NE often prevents the treatment of the disease. Antibiotics have been commonly used worldwide as growth promoters and for prophylactic treatment of *C. perfringens*-induced NE in poultry. However, with the European ban on antibiotics (feed additives regulation 1831/2003/EC), which took effect in January 2006, alternatives to antibiotics became essential in order to prevent NE occurrence and the consequent economic losses for the poultry industry. Preventive treatments can take the form of actions on predisposing factors, such as coccidiosis prevention, diet modifications, or improving overall cleanliness and hygiene. Alternatively they can directly target the causal agent of the disease by controlling the proliferation, colonization and persistence of virulent strains of *C. perfringens* or interfering with virulence and pathogenicity factors (Figure 1). *C. perfringens* infections can be reduced or abolished by using natural feed additives, such as probiotics (yeasts or bacteria), plants (Engberg et al., 2012), molecules of plant origin [for example, essential oils (Mitsch et al., 2004; Timbermont et al., 2010) or Annatto extracts (Galindo-Cuspinera et al., 2003)], organic acids (Geier et al., 2010; Timbermont et al., 2010), enzymes (Jackson et al., 2003; Engberg et al., 2004), lysozyme (Liu et al., 2010), or molecules of microbial origin, such as yeast extract and antimicrobial peptides (Figure 1). Here we give an overview of these preventive treatments, by focusing on micro-organisms and molecules or products of microbial origins that affects *C. perfringens* growth and pathogenicity.

## Feeding “live” bacteria and yeasts

Supplementation of the broilers' diet with one or several beneficial bacteria has proven to be efficient to prevent the overgrowth of pathogens and the subsequent diseases. Several bacterial strains have been shown to increase broiler chickens performance (health, weight gain, feed conversion) and to prevent or reduce the incidence of diseases caused by pathogenic bacteria (reviewed by Patterson and Burkholder, 2003; Lutful Kabir, 2009; Chaucheyras-Durand and Durand, 2010). Probiotics, or direct-fed microbials, and competitive exclusion (CE) cultures are thus commonly used in broiler farms. There are several commercially available products that have been shown to be efficient against *C. perfringens* and NE in poultry (Table 1).

**Table 1:** **Examples of commercially available microbial feed additives for NE prevention in poultry**.

**Product**	**Company**	**Composition**	**Origin**	**Activity**	**Selected references**
Aviguard®	MSD Animal Health	Over 200 bacterial species	Healthy, adult chickens	Competitive exclusion	Hofacre et al., 1998
BROILACT®	Nimrod Veterinary products	Complex mixture of bacteria	Intestine of a normal adult fowl	Competitive exclusion	Kaldhusdal et al., 2001
PoultryStar®	Biomin	6 bacterial species and 1 prebiotic (FOS)	Unknown	Competitive exclusion	McReynolds et al., 2009
MSC™	Continental Grain Co.	Bacteria	Caeca and caecal sections	Competitive exclusion	Craven et al., 1999
Finelact™	QTI Animal Health	*L. reuteri*	Live, healthy chicken	Probiotic	Tested in a field trial (manufacturer's product data)
FloraMax® B-11	Pacific Vet Group, USA	11 lactic acid bacteria and inactivated *Saccharomyces cerevisiae*	Poultry intestine	Probiotic	Layton et al., 2013
NuPro®	Alltech Inc.	Yeast extract	Yeast	Immunostimulation, antimicrobial activity	Thanissery et al., 2010
SafMannan®	Phileo Lesaffre Animal Care	Yeast Extract	*S. cerevisiae*	Immunostimulation, antimicrobial activity	Abudabos and Yehia, 2013

### Probiotics

A probiotic is defined as “a live microbial food supplement that beneficially affects the host by improving the intestinal microbial balance” (Fuller, 1999). Indeed, probiotics can interact with the host to improve immunity and intestinal morphology or stimulate the metabolism, thus reducing the risk of infection by opportunistic pathogens. Probiotic bacteria have also been shown to produce molecules with antimicrobial activities, such as bacteriocins, that target specific pathogens, or even inhibit the adhesion of pathogens or the production of pathogenic toxins (Joerger, 2003; Pan and Yu, 2014). Moreover, beneficial bacteria can act as competition against pathogenic strains within the host. The concept of competitive exclusion will be discussed further below. For the purpose of this section, we have chosen to focus on strains that were shown to have an effect on NE incidence in poultry, through a targeted antagonistic activity against *C. perfringens*.

A large number of studies described the isolation of microorganisms with anti-*C. perfringens* activity *in vitro* (Table 2). Most of these strains belong to the genera *Bacillus* and *Lactobacillus*. Very few reports discussed the deployment of live yeasts with antagonistic activity against *C. perfringens*, their use in NE prevention being limited to inactivated yeast or yeast extract (Tables 1, 2).

**Table 2:** **Examples of probiotic strains with anti-***C. perfringens*** activity ***in vitro*** and ***in vivo*****.

**Strain**	**Origin**	**Anti-Cp activity *in vitro***	**Anti-Cp activity *in vivo* (poultry model)**	**Mode of action**	**References**
***Bacillus***					
*Bacillus cereus* 8A	n.s.	+	n.t.	Bacteriocin	Bizani and Brandelli, 2002
*Bacillus licheniformis*	Broiler GI tract	+	n.t.	n.s.	Barbosa et al., 2005
	n.s.	n.t.	+	Spores	Knap et al., 2010
*B. pumilus*	Broiler GI tract	+	n.t.	n.s.	Barbosa et al., 2005
*B. subtilis*	Broiler GI tract	+	n.t.	n.s.	Barbosa et al., 2005
	Porcine intestine	+	n.t.	Bacteriocin	Klose et al., 2010
	Healthy chicken GI tract	+	+	Protein	Teo and Tan, 2005; Jayaraman et al., 2013
	n.s.	−	+	Spores	La Ragione and Woodward, 2003
***Enterococci***					
*E. faecium*	Porcine intestine	+	n.t.	Lactate and H_2_O_2_	Klose et al., 2010
	Fermented food	+	n.t.	3,000 Da BLIS	Chen et al., 2007
	Broiler GI tract	+	n.t.	Enterocin A/B	Shin et al., 2008
	n.s.		+	n.s.	Cao et al., 2013
*E. faecalis*	Human	+	n.t.	Bacteriocin	Bottone et al., 1974
	Human	+	n.t.	n.s.	Stark, 1960
	n.s.	−^a^	+	Toxin inhibition	Fukata et al., 1991
*E. durans*	Human	+	n.t.	n.s.	Stark, 1960
***Bifidobacteria***					
*B. animalis ssp lactis*	Commercial strain	+	n.t.	NS molecule	Schoster et al., 2013
*B. infantis*	n.s.	+	n.t.	n.s.	Gibson and Wang, 1994
*B. thermoacidophilum*	Porcine intestine	+	n.t.	Lactate and H_2_O_2_	Kim et al., 2007; Klose et al., 2010
***Lactobacilli***					
*Lactobacillus* sp.	Chicken feces	+	+	n.s.	Gérard et al., 2008
*L. acidophilus*	n.s.	−^a^	+	Toxin inhibition	Fukata et al., 1991
*L. amylovorus*	Porcine intestine	+	n.t.	Lactate and H_2_O_2_	Kim et al., 2007; Klose et al., 2010
*L. animalis*	Dog feces	+	+^b^	n.s.	Biagi et al., 2007
*L. fermentum*	Porcine epithelium	+	n.t.	Toxin inhibition	Allaart et al., 2011
	Reference strain	n.t.	+^c^	n.t.	Cao et al., 2012
*L. johnsonii* FI9785	Poultry	−	+	n.t.	La Ragione et al., 2004
*L. mucosae*	Porcine intestine	+	n.t.	Lactate and H_2_O_2_	Klose et al., 2010
*L. plantarum*	Commercial strain	+	n.t.	BS molecule	Schoster et al., 2013
*L. reuteri*	Porcine intestine	+	n.t.	Lactate and H_2_O_2_	Kim et al., 2007; Klose et al., 2010
*L. salivarius*	Chicken intestine	+	n.t.	Lactate and H_2_O_2_	Kim et al., 2007; Klose et al., 2010

a *Active against toxin production*.

b *Active in a canine model*.

c *Reduced inflammation*.

*n.s., not specified; n.t., not tested; BLIS, bacteriocin-like inhibitory substance; NS, narrow spectrum; BS, broad spectrum*.

#### Yeasts

Despite being under-represented in the literature as anti-*C. perfringens* agents, yeasts are known to have antimicrobial properties, which were recently reviewed (Hatoum et al., 2012). In addition the cell wall is, for many types of yeast, rich in beta-glucans, which have immunomodulatory properties (Novak and Vetvicka, 2008). On top of the beneficial effects they have on the host, yeasts can constitute a protection against pathogens by (i) producing mycocins, (ii) secreting enzymes that degrade bacterial toxins, (iii) preventing adhesion to epithelial cells, or (iv) by acting as a competitive exclusion agent (reviewed by Hatoum et al., 2012). For example, *Debaromyces hansenii* secretes molecules with anti-*C. butyricum* activity (Fatichenti et al., 1983), and *Saccharomyces boulardii* secretes a serine protease that inhibits the action of *C. difficile* toxins *in vivo* and *in vitro* (Castagliuolo et al., 1996, 1999).

Field trials using live *S. boulardii* as feed additives obtained positive results on performance and intestinal health improvement in healthy chickens (Rajput et al., 2013) and in chickens infected with *Salmonella* Enteritidis (Gil de los santos et al., 2005). Moreover, another study by the same authors showed that using a recombinant strain of *Pichia pastoris* carrying the gene coding for the *C. perfringens* α toxin, not only improved broiler chickens performance, but also increased the secretion of anti-*C. perfringens* antibodies (Gil de los santos et al., 2012). It would be interesting to test the effects of these strains on the mortality and *C. perfringens* counts in *C. perfringens*-induced NE challenged birds. It is also worth noting that two fungi of the genus *Fusarium* were reported to produce mycocins with anti-*C. perfringens* activity (enniatin B of *F. tricinctum* and the beauvericin of *F. proliferatum*), which were active at low concentrations (20 μg/ml and 0.1 μg/ml, respectively; Meca et al., 2010, 2011).

#### Bacillus species

Several strains of *Bacillus* have been shown to have antagonistic activity against *C. perfringens* (Table 2). In most studies, the activity was linked to the production of bacteriocins. Indeed, within the *Bacillus* genus, several species are known to produce bacteriocins and antimicrobial peptides (Stein, 2005; Lee and Kim, 2011; Mongkolthanaruk, 2012; Cochrane and Vederas, 2014). For example, *B. thuringiensis* produces thuricin which is active against *C. difficile* (Rea et al., 2010).

The antagonistic species described in the literature include *B. cereus, B. licheniformis, B. pumilus*, and *B. subtilis*, which was the most represented. In a study involving over 200 *Bacillus* strains isolated from broiler feces, Barbosa et al. (2005) identified several species (*licheniformis, pumilus, subtilis*) with activity against *C. perfringens in vitro* (Barbosa et al., 2005). A *Bacillus cereus* strain, isolated from a soil sample in Brazil, also showed antagonism against *C. perfringens*. The activity of the strain was ascribed to the production of a bacteriocin during the exponential phase of growth (Bizani and Brandelli, 2002). Teo and Tan (2005) isolated *B. subtilis* strain SP6 and showed that it had anti-*C. perfringens* activity *in vitro* (Teo and Tan, 2005). The authors identified the molecule responsible for the antagonistic activity as a 960–983 Da molecule of proteinacious nature that was highly heat-stable (Teo and Tan, 2005). The same strain was used in a NE challenge field trial involving 216 chicks and was shown to reduce mortality by half, to improve intestinal health (as measured by villi length) and to significantly reduce *C. perfringens* counts (Jayaraman et al., 2013).

The supplementation of animal feed with *Bacillus* spores was also tested and proven to be an efficient alternative to the use of antibiotics. When 20 day old chicks, inoculated with low amounts of *C. perfringens*, were given a single dose of 10^9^ *B. subtilis* spores, colonization and persistence of *C. perfringens* were abolished, although the *B. subtilis* strain alone was shown to be unable to affect *C. perfringens in vitro* (La Ragione and Woodward, 2003). In another field trial, Knap et al. (2010) tested the effect of adding *B. licheniformis* spores to the diet, but used larger amounts and for longer periods of time (Knap et al., 2010). They observed increased performance and reduced mortality in the group of chicks treated with the spores.

#### Enterococci

A strain of *E. faecium* when fed to chicks on day of hatch was shown to reduce numbers of *C. perfringens* along with other pathogens after 28 days, and concomitantly to increase the counts of lactic acid bacteria (*Lactobacilli* and *Bifidobacteria*) (Cao et al., 2013). Klose et al. (2010) tested a number of *Enterococcus* strains, isolated from various animals, for their antagonism against *C. perfringens* and found that almost all had anti-*C. perfringens* activity, which could be attributed to the production of acids and hydrogen peroxide (Klose et al., 2010). *Enterococci* are known to produce a wide-range of bacteriocins, called enterocins, which are active against Gram-positive and Gram-negative bacteria (Franz et al., 2007). Shin et al. (2008) isolated a strain of *E. faecium* from broiler intestines that was active against *C. perfringens in vitro*, and identified the antimicrobial molecules as enterocins, with high homology to enterocins A and B (Shin et al., 2008). Strains of *E. faecalis* were also active against *C. perfringens in vivo* (Stark, 1960; Bottone et al., 1974; Fukata et al., 1991) (Table 2). One strain even prevented *C. perfringens* proliferation *in vivo* and inhibited α toxin production *in vitro* (Fukata et al., 1991). However, the potentially pathogenic nature of *E. faecalis* could prevent its use as a probiotic feed additive.

#### Lactic acid bacteria

Lactic acid bacteria (LAB) are also very good probiotic candidates, as they display antimicrobial activities, but also have beneficial effects for the host. Cao et al. (2012) showed that adding *L. fermentum* I.2029 to the diet of young chicks reduced the occurrence of *C. perfringens*-induced ileal lesions and inflammation. However, the effect on *C. perfringens* numbers was not measured in this study. Cao and colleagues also showed that the addition of the probiotic stimulated the host immune system, as seen by increased levels of cytokine expression, measured by real-time PCR (Cao et al., 2012). Many LAB isolates have exhibited anti-*C. perfringens* activity *in vitro* (Table 2). For example, in a screening experiment involving 104 *Lactobacillus* strains isolated from geese feces, 84 strains were active against *C. perfringens* (Dec et al., 2014). Related to that, Kim et al. (2007) isolated several LAB (*Lactobacillus* and *Bifidobacterium*) from pig intestines that had antagonistic action against *C. perfringens*, with an *L. amylovorus* strain presenting properties amenable to be a potential probiotic candidate (Kim et al., 2007). The antimicrobial action of LAB is often attributed to the secretion of bacteriocins or the production of organic acids. Schoster et al. (2013) tested the inhibitory activities of several commercial strains against *C. perfringens in vitro*. These authors identified 2 LAB strains, *L. plantarum*, and *B. animalis* spp. lactis, with antagonism against reference strains, but also against clinical isolates (Schoster et al., 2013). The *L. plantarum* strain was active against Gram negative and Gram positive strains, whereas the *Bifidobacterium* isolate had a narrower spectrum of activity (Schoster et al., 2013).

The ability of a potential probiotic strain to survive in the host without affecting the bacterial balance or the beneficial flora is of major importance. In this regard, the *in vitro* studies that report the isolation of potential probiotic strains almost always test the strain for their ability to survive in the host and exert their action *in vivo*, through assessment of acid and bile resistance, auto-aggregation and adhesion to epithelial cells. A strain of *Lactobacillus* species that reduced *C. perfringens* numbers in chickens was shown not to affect the commensal flora (Gérard et al., 2008). In a trial conducted by La Ragione et al. (2004), 20 day old chicks were fed a strain of *L. johnsonii* (FI9785). This strain was able persist in the chicks for the duration of the experiment, caused a reduced colonization by *C. perfringens* after 15 days, although no direct anti-*C. perfringens* activity was evident *in vitro* (La Ragione et al., 2004). Layton and colleagues tested the efficacy of the probiotic FloraMax-B11 (FM-B11), which consists of several LAB strains (Table 1) on chicks challenged with *E. maxima, S. typhimurium* and *C. perfringens* (Layton et al., 2013). The chicks started receiving the probiotic on day 14 and were infected 7 days later. After 10 days, they observed a high reduction of mortality in the group treated with FM-B11, along with reduced intestinal lesions and *C. perfringens* counts. Furthermore, FM-B11 was shown to be active against *C. perfringens in vitro* (Layton et al., 2013). Allaart et al. (2011) described an example of an antagonistic action that targeted pathogenicity *via* the inhibition of toxin production. A probiotic strain of *L. fermentum* of porcine origin negatively regulated the production of the β2 toxin by *C. perfringens*, without affecting cell growth (Allaart et al., 2011). The action of *L. fermentum* on β2 toxin production appeared to occur at the transcription level and was exerted through an effect on the environmental pH. The exact mechanism is however still unclear and it is not known whether the same effect would be observed *in vivo* (Allaart et al., 2011).

### Competitive exclusion

The concept of competitive exclusion (CE) was originally described by Nurmi and Rantala in 1973, when feeding young chicks with bacteria isolated from a healthy adult chicken prevented colonization by *Salmonella infantis* (Nurmi and Rantala, 1973; Rantala and Nurmi, 1973). The exact mechanisms of action of CE remain unclear. However, it is now well known that implanting a “healthy” flora in the early days of life accelerates the establishment of the intestinal flora and creates a competition for nutrients within the intestine, thus preventing colonization by pathogens (Joerger, 2003; Schneitz, 2005). Moreover, the beneficial effects can be due to the intrinsic properties of the bacteria composing the mixture, as described above in the “Probiotics” section. CE products were initially described against *Salmonella* in chickens. Since then several studies focusing on the effect of CE to prevent *C. perfringens*-associated CE have been published, and several commercial products with proven effects on *C. perfringens*-induced NE are available (Table 1).

The first reports discussing the use of caecal contents from healthy chickens to prevent NE in young chicks date from the early 80 s. Barnes et al. (1980) described experiments in which 1-day-old chicks were fed caecal samples from healthy hens, containing, among others, several *Lactobacillus, Streptococcus faecalis, S. faecium* (now called *Enterococcus faecalis* and *E. faecium*) and *Bacteroides hypermegas* (Barnes et al., 1980). After 3 days, they observed a reduction in the number of *C. perfringens*, ranging from 100 to 1000 times lower in treated animals. Since then, several reports have been published, reporting a globally better intestinal health, a reduction in the number of *C. perfringens* and lower mortality, after administration of a CE product. The composition and efficacy of commercialized CE cultures have been the focus of several studies (Table 1). Elwinger and colleagues showed that the use of Broilact® reduced the mortality and occurrence of NE, with less *C. perfringens* in the caecum of animals in the treated group (Elwinger et al., 1992). Another field trial involving Broilact® was performed by Kaldhusdal et al. (2001) where chicks were sprayed with this CE product on the day of hatch. They observed a reduction in *C. perfringens* counts, lower incidence of NE and NE-associated gut lesions, with a most significant effect observed in the early weeks of life (Kaldhusdal et al., 2001). Another research group tested the efficacy of MSC™ (for Mucosal Starter Culture) which is another commercial product consisting of bacteria isolated from the gut of a healthy chicken (Craven et al., 1999). They used several virulent strains of *C. perfringens* to challenge the animals and fed them MSC in their first 3 weeks of life; interestingly, although they did not observe an effect on the number of *C. perfringens*, they detected less enterotoxin in the treated group compared to the control one, suggesting a selection for less virulent *C. perfringens* strains. In another trial, they additionally fed the birds a diet known to predispose them to NE, which resulted in a reduction in toxins present and but also in the numbers of *C. perfringens* (Craven et al., 1999). Hofacre et al. (1998) performed a trial involving 900 chicks which were challenged with *C. perfringens* and *E. acervulina* when 14 days old. The chicks were treated with Aviguard®, two other CE products and one probiotic 3 days later. They observed reduced mortality in the chickens treated with CE cultures compared to the ones that only received the probiotic. Moreover, the CE-treated animals had a reduction in lesions to the intestinal mucosa and displayed overall increased feed conversion (Hofacre et al., 1998). The same researchers later tested a different CE product containing *L. acidophilus, L. plantarum, E. faecium* and *Pediococcus acidilactia* (All-Lac XCL, Alltech) in combination with a prebiotic (MOS, Alltech) and observed a reduction of mortality by half compared to the untreated animals, with effects comparable to those of bacitracin (Hofacre et al., 2003). McReynolds et al. (2009) also tested the effects of CE cultures in association with a prebiotic containing essential oils and fructo-oligosaccharides (FOS) on chicks given an immunosuppressant vaccine, inoculated with *C. perfringens* and in dietary conditions favorable to NE development (McReynolds et al., 2009). Both the prebiotic and the CE cultures led to reduced *C. perfringens* counts, a reduction in the intestinal lesions and lower mortality (McReynolds et al., 2009). Overall, the use of CE cultures in combination with other products, probiotics and prebiotics that have a more targeted action on *C. perfringens* appears to be more effective to prevent NE occurrence in poultry.

## Molecules of microbial origin

### Prebiotics

Prebiotics are additives that will stimulate the commensal flora and enhance the beneficial effects of probiotics within the host and are mostly indigestible oligosaccharides (Patel and Goyal, 2012). Numerous molecules have been described, with mannan-oligosaccharides (MOS) being the main prebiotic of microbial origin. MOS are components within the yeast cell wall and constitute the main active ingredient of yeast extract (YE) for disease control. They are often used as feed additives in broiler diets (Table 1) where they have been shown to improve intestinal health and immune response, and also inhibit pathogen colonization by reducing adhesion. The addition of MOS to broiler feed was shown to improve overall performance as measured by productivity and weight gain (Fowler et al., 2015). Thanissery and colleagues tested the effect of adding 2% yeast extract (NuPro, Alltech) to broiler feed, for the first 10 days of life, before a challenge with type A *C. perfringens* strains (Thanissery et al., 2010). Overall, animals treated with NuPro had fewer lesions in the duodenum compared to the untreated ones, to a degree comparable to the group treated with the antibiotic bacitracin (Thanissery et al., 2010). The *C. perfringens* counts were one to two logs lower in the treated groups; however, the difference was not statistically significant; the authors suggested the use of NuPro for longer periods in order to improve its efficiency. Recently, Abudabos and Yehia (2013) tested another commercial yeast extract additive, Saf-Mannan, in a field trial for its ability to protect broiler chickens against NE (Abudabos and Yehia, 2013). They performed a *C. perfringens* challenge on 16 days-old birds that were fed 0.05% Saf-Mannan since hatching, and compared their performance, gut health and *C. perfringens* counts on day 30. The chicks that were given the yeast extract showed overall better intestinal health (based on villi height) and had improved performance (measured by body weight gain and feed conversion ratio), which are consistent with the known beneficial effect of yeast extract on broiler performance. Moreover, the animals treated with Saf-Mannan had a 5 log reduction of *C. perfringens* numbers in the small intestine in comparison to the untreated animals (Abudabos and Yehia, 2013). However, caution is required when discussing the anti-*C. perfringens* of MOS or YE, as the antagonistic effect seems to be highly variable and dependent on a number of variables, such as dose, length of treatment or even diet (Biggs et al., 2007; Jacobs and Parsons, 2009; Kim et al., 2011).

### Bacteriocins

Bacteriocins are small ribosomally synthesized antimicrobial peptides that are produced by a large number of bacteria. They are classified based on their size, structure and post-translational modifications (Cotter et al., 2013). One of the main benefits of the use of bacteriocins is that some of them present a highly specific antimicrobial activity, so that they can be used to treat specific infections without altering the commensal gut flora. As discussed previously, the action of many probiotic strains is exerted through the secretion of bacteriocins (Table 2). Several examples of well-described bacteriocins with beneficial effects for broilers can be found. These include pediocin A, produced by *Pediococcus pentosaceus*, and divercin of *Carnobacterium divergens*, which were shown to improve broiler performance in a field trial (Grilli et al., 2009; Józefiak et al., 2012) as well as the well-characterized nisin produced by *Lactococcus lactis* that was shown to affect *C. perfringens* cells and spores *in vitro* (Udompijitkul et al., 2012). A strain of *Ruminococcus gnavus*, isolated from an healthy human feces, was shown to produce a 2.6 kDa bacteriocin (Ruminococcin A, class IIA lantibiotics) that was highly active against *C. perfringens in vitro* (MIC = 75 μg/ml) (Dabard et al., 2001). However, ruminococcin A is poorly expressed *in vivo* as tested in *R. gnavus*-inoculated rats, a potential limit on its usefulness (Crost et al., 2011). In contrast, another bacteriocin (Ruminococcin C) identified by the same research group, which was active against *C. perfringens* with a MIC of 40 μg/ml, was expressed *in vivo* (Crost et al., 2011). Lee et al. (2011a) identified an anti-*C. perfringens* lantibiotic produced by *Bifidobacterium longum* that had a broad range of inhibition. Sharma and colleagues identified a strain of *Brevibacillus borstelensis* with anti-*C. perfringens* activity that was associated with a thermostable bacteriocin-like inhibitory substance (BLIS) of 12 kDa, which was active under the physiological conditions expected in the GI tract (Sharma et al., 2014).

The use of purified bacteriocins or the producing strains as feed additives represents a realistic alternative to conventional antibiotics. Thorough characterizations are required, however, to confirm the synthesis and the integrity of the molecule in GI tract conditions. Moreover, the potential development of resistance in the *C. perfringens* target organism needs to be taken into account.

## Bacteriophages

Bacteriophages are highly species-specific viruses that infect and kill bacteria. Upon replication within the bacterial cell, phages produce endolysins, which target peptidoglycans and lyse the bacterial cell wall, freeing the phages and allowing them to spread to other cells (Nakonieczna et al., 2015). Phages were first discovered and described a century ago (Twort, 1915; d'Hérelles, 1917). Phage therapy was widely used to treat bacterial infections until the 40 s, and has seen a recent upsurge in interest with the growing need for alternatives to antibiotic treatments to treat diseases caused by antibiotic-resistant bacteria.

Many bacteriophages of *C. perfringens* have been described and sequenced (Morales et al., 2012; Seal et al., 2012; Volozhantsev et al., 2012), including several that were isolated from strains of poultry origin and that had specific anti-*Clostridium* activity (Zimmer et al., 2002a; Seal et al., 2011; Volozhantsev et al., 2011; Seal, 2013). The use of bacteriophages to limit *C. perfringens* infection has proven efficient in field trials. For example, Miller et al. (2010) showed that feeding broilers with a mixture of six bacteriophages reduced mortality in an NE challenge by over 90% and improved overall performance assessed as weight gain and feed conversion (Miller et al., 2010).

A number of studies focused on the use of bacteriophage endolysins as antimicrobials, rather than the phage itself (Zimmer et al., 2002b; Tillman et al., 2013; Gervasi et al., 2014a; Swift et al., 2015). The use of phage proteins instead of bacteriophages eliminates complications that can arise with phage therapy. Indeed, several studies have described bacteria becoming resistant to phage infection, by developing mechanisms to prevent the entry of the phage in the cell or by degrading the injected DNA (Nobrega et al., 2015). A purified recombinant endolysin of bacteriophage ϕ3626, isolated from a type strain of *C. perfringens*, was shown to have lytic activity against over 40 strains of *C. perfringens*, without affecting other *Clostridium* species or species of different genera, such as *Lactobacillus, Enterococcus* or *Bacillus* (30 and 34 strains tested respectively) (Zimmer et al., 2002a,b). Recently, a modified endolysin was shown to be active against *C. perfringens* even at high temperatures, making it a suitable candidate as an antimicrobial additive for NE prevention (Swift et al., 2015). Another research group characterized the endolysin CP25L, isolated from a *C. perfringens* bacteriophage, which was active against *C. perfringens in vitro* (Gervasi et al., 2013, 2014a). The authors were able to over-express the enzyme in a modified *L. johnsonii* strain, strain which was discussed earlier in this review as active against *C. perfringens in vivo* (La Ragione et al., 2004; Gervasi et al., 2014a,b). This strain was able to survive in GI tract-like conditions, but the expression of the endolysin and the control of *C. perfingens* growth in co-culture were inconsistent (Gervasi et al., 2014b). The use of probiotic strains to deliver antagonist molecules within the GI tract is a promising alternative; however, the application can be problematic. Indeed, it is hard to predict the behavior of the molecule *in vivo* and many factors can interfere with its synthesis by affecting the producer strain.

## Vaccination against *C. perfringens*

A large number of trials tested the efficacy of broiler vaccination as a prophylactic treatment against *C. perfringens*-induced NE. For the purpose of this review, we will limit this section to an overview of the recent advances regarding vaccines against *C. perfringens*. The reader is also directed to a recent review by the Van Immerseel lab (Mot et al., 2014). Several strategies have been used to vaccinate broilers against *C. perfringens* to include use of live bacteria or inactivated toxins. Vaccines can be delivered by spraying chicks upon hatching, by addition to the feed or the drinking water, or even injected *in ovo* (Sharma, 1999; Muir et al., 2000; Mot et al., 2014). Vaccination using non-virulent *C. perfringens* strains have proven to be inefficient, and it has been shown that strains used in vaccines need to remain mildly virulent. Thompson et al. (2006) showed that strains with a mutation in the gene coding for the α toxin that were still virulent (but less than the wild-type) were able to protect chickens against NE, whereas an avirulent strain of *C. perfringens* did not have any immunizing effects (Thompson et al., 2006).

Several trials have shown that chickens could be protected against *C. perfringens*-induced NE by injection with inactive and active toxins (Kulkarni et al., 2007; Jang et al., 2012) and antigenic proteins (Jiang et al., 2009). Since the discovery of its role in NE, the NetB toxin has been intensively studied with regards to vaccination, with some promising results (Fernandes da Costa et al., 2013; Keyburn et al., 2013a,b).

## Conclusions and perspectives

A number of studies have now shown that the use of live micro-organisms and molecules produced by microbes represent potential alternatives to the use of conventional antibiotics for the prevention of *C. perfringens*-induced NE in broiler chickens. Although a large number of probiotic bacterial strains with *C. perfringens* antagonism have been described, studies and trials using live yeasts are surprisingly sparse and in our opinion warrant further investigations. Antimicrobial molecules, such as bacteriocins or phage endolysins, are also good candidates for new antimicrobials. Research for new antimicrobials is, however, limited by regulations and applicability and mainly focuses on the use of GRAS micro-organisms, with restricted use of genetically modified organisms (GMOs). Nanoparticles could also be used as vectors for delivery of these new antimicrobial molecules, thus avoiding the expression and regulatory issues that arise with the use of live cells and GMOs.

It is difficult to identify a single “ideal” solution within this wealth of options for NE disease control. Several microbes and molecules of microbial origins, some already available commercially, represent promising agents that conceivably could be used in conjunction with one another to formulate highly effective synergic antimicrobials. For example, a product consisting of a CE culture, a probiotic strain producing a targeted anti-*C. perfringens* molecule and a prebiotic product would constitute a robust formulation that could prevent the overgrowth of *C. perfringens in vivo* and maintain a healthy GI tract flora at the same time.

Several criteria must be taken into account when developing feed additives or preventive treatments for the animal industry. The financial cost of the product is a major criterion, especially for small animals with low market value, like broiler chickens. A thorough genetic characterization of candidate strains is essential in order to confirm the safety of the bacterial strain and ensure the lack of virulence and antibiotic resistance genes. The chosen molecule or strain must be able to stay active in the host and withstand industrial treatments. One must also keep in mind that the GI tract is a highly complex environment with numerous bacterial species that can affect the efficacy of these antimicrobials, perhaps in a different manner from one animal to another. Moreover, *C. perfringens* and other bacteria are highly adaptable micro-organisms. It is thus of high importance to develop and use products in a rational manner in order to avoid the appearance of strains resistant to these novel antimicrobials, as has occurred with conventional antibiotics.

## Author contributions

DC, RD, EA, and DD contributed to the conception and the design of the review and researched and wrote the review.

### Conflict of interest statement

The authors declare that the research was conducted in the absence of any commercial or financial relationships that could be construed as a potential conflict of interest.
